# Feasibility and testing outcomes of task-shared implementation of advanced HIV disease point of care tests in Beira (Mozambique) and Kinshasa (DRC)

**DOI:** 10.1371/journal.pone.0339469

**Published:** 2026-02-09

**Authors:** Zibusiso Ndlovu, Ruvimbo Nhandara, Kiyara Govender, Fingani Mpande-Nyasulu, Emmanuel Kabwe, Antonio Flores, Calorine Mekiedje, Laban Kyembe, Christine Bimansha, Tania Carmilla Tomas, Leticia Penna, Mariana da Silva, Micheque Jose Tauro, Ana Jossias Bonde, Luisa Rita Miguel, Pedro David Manusso, Gisele Mucinya, Yvonne Nzomukunda, Rollin Ndombe, Richard Ingwe Chuy, Aimé Mboyo, Tom Ellman, Geoffrey Fatti

**Affiliations:** 1 Medecins Sans Frontières (MSF), Beira, Mozambique; 2 Division of Epidemiology and Biostatistics, Department of Global Health, Faculty of Medicine and Health Sciences, Stellenbosch University, Cape Town, South Africa; 3 Southern African Medical Units (SAMU), Medecins Sans Frontières, Cape Town, South Africa; 4 Faculty of Medicine, King Mongkut’s Institute of Technology Ladkrabang, Bangkok, Thailand; 5 Kazan (Volga region) Fedaral University, Kazan, Russia; 6 Ministry of Health Mozambique, Maputo, Mozambique; 7 Medecins Sans Frontières (MSF), Kinshasa, Democratic Republic of the Congo; 8 National HIV/AIDS and STI Control program of the Democratic Republic of the Congo, Kinshasa, Democratic Republic of the Congo; Monash University, AUSTRALIA

## Abstract

**Introduction:**

Mortality from advanced HIV disease (AHD) remains high and current strategies to promptly test people eligible for AHD screening, are insufficient. Task sharing for point of care (POC) testing utilizing lay health workers (LHW) is recommended, however it is marginally practised in many countries. This study sought to describe the feasibility and testing outcomes of task-shared implementation of the AHD POC diagnostic tests utilizing LHW and professional health care workers (HCW).

**Methods:**

This was a cross-sectional mixed-methods implementation study in seven primary and three secondary health facilities, in Mozambique and Democratic Republic of Congo (DRC). From March to November 2022, consenting HIV positive adults eligible for AHD screening, were offered Visitect CD4 lateral flow assay (LFA), and or subsequently urinary *Mycobacterium tuberculosis* lipoarabinomannan antigen (TB LAM) and cryptococcal antigen (CrAg) tests. The primary study outcome was the proportion of testers (LHW and HCW) who found it ‘easy’ to integrate the 3 POC tests within their routine work together with their opinions on the value of task shared AHD POC testing.

**Results:**

A total of 1542 patients were screened for AHD by 35 LHW (34 counsellors, and 1 lay educator) and 45 professional HCW (28 nurses and 9 clinical officers and 8 doctors). In the study period, LHW conducted a median number of 27 [IQR: 16–34] Visitect CD4 LFA tests, whereas nurses, clinical officers together with doctors conducted 19 and 11 respectively. Visitect CD4 LFA increased CD4 testing by 10.7% in Munhava (Beira) and 22.9% in CHK (DRC), complementing existing CD4 testing instruments. Among testers who completed the feasibility survey, nearly sixty percent of testers (25/42; LHW in particular) found it easy to integrate AHD POC testing within their routine workflow.

The prevalence of AHD was 39.2% (604/1542). A total of 34% (146/430) and 5.4% (22/407) of patients tested positive for urine TB LAM and plasma CrAg respectively. Of these, 82.2% (120/146) and 36.4% (8/22) had a documented therapeutic intervention. The median time for completing the Visitect CD4 LFA and conveying the results to the clinician was 59 minutes [IQR: 48–71].

**Conclusion:**

Task-shared integrated testing for AHD at POC among LHW and professional HCW, is feasible and can improve access to AHD testing. However, as POC testing responsibilities become shared, documentation of testing activities could increase in complexity and can be easily fragmented, especially when there is limited supervision. Nevertheless, LHW are well suited for POC testing due to limited availability and higher clinical workload of other HCW.

## Introduction

Point-of-care testing (POCT) refers to any diagnostic test administered near or at the site of a patient where the specimen is collected as opposed to a conventional clinical laboratory setting. POCT can ensure quicker access to health services [1–4] and availability of POCT is set to be on the increase due to increasing advancements in in-vitro diagnostic medical technologies and patient-centred care [4]. Despite the sustained investments in the development of innovative POC devices, POCT at primary health care (PHC) facilities without laboratories, lacks the necessary support for testing and responsibility for quality assurance. This danger lacks the warranted urgency and planning for solutions, and it currently limits the possible impact of POCT [5,6].

The proportion of people with HIV (PWH) presenting with advanced HIV disease (AHD) at antiretroviral therapy (ART) initiation remains high (30%) [7–10] and in the year 2023, an estimated 630,000 PWH died from AIDS-related causes [8]. Evidence from population-based survey data shows that nearly two-thirds of people with AHD are on ART [11]. Majority of countries with high AHD burden rely on limited centralized and POC CD4 cell count testing instruments, mostly used in a hub-and-spoke model [12]. Operability of a substantial number of these POC CD4 instruments is a concern as some are nearing the end of their expected lifespan [13]. Coverage of CD4 testing among patients eligible for AHD screening remains very low in most sub-Saharan Africa and could even get lower as manufacturers of major POC CD4 cell count testing instruments, exited the market [14] due to low testing volumes. Across six countries in southern Africa, CD4 testing at ART initiation declined from 78% in 2008 to only 38% in 2017 [15] and programmatic data suggest that up-to 70% of eligible PWH are not accessing the critical AHD screening and diagnostic package [15–18]. Current strategies to promptly screen people eligible for AHD are insufficient.

POCT for AHD could complement laboratory-based testing, while also allowing for the immediate return of results and expedited clinical management. However, general use of POC tests for AHD (particularly in health facilities with no laboratories) largely remains the responsibility of frontline health care workers (HCWs). The resultant trade-off with their other roles, usually sees POC tests not used to maximal benefit of patients [3,4]. WHO strongly recommended task sharing of POCT with non-laboratory personnel [19] as a pragmatic response to HCW shortages. Task sharing is the rational re-allocation of tasks from professional HCWs to trained lay health workers (LHWs) [20]. LHW are personnel who perform functions related to healthcare delivery but do not possess formal professional or paraprofessional education. They typically receive training tailored to their specific role [20]. Numerous studies including systematic reviews and meta-analysis have shown that LHWs, with adequate training, can reliably conduct POCT services [1-4,21–27]. LHW in the studies ranged from trained counsellors, health diagnostic assistants, phlebotomists, microscopists, and health surveillance assistants; and reasons for task sharing ranged from HCW shortages to the need to expand and decentralize access to health services. However, implementation of task sharing for POCT with LHW in national programs has been marginal and limited to protocols of vertical disease programs, and this restricts LHW to narrow testing functions [4,28].

Even though numerous studies have shown that task sharing or shifting with LHWs is non-inferior to laboratory-based testing [1–4,21–27], there is a scarcity of studies that explore feasibility of LHW-led task-shared integrated POCT for AHD, specifically using the recommended three minimal tests (CD4 cell count, urine TB LAM and CrAg). Such studies could inform country-level considerations about the prospects of task shared integrated POCT for AHD and challenge persistence of rigid professional boundaries together with siloed vertical disease program implementation of task-shifted POCT.

The objective of this study was to explore feasibility of task-shared integrated POCT for AHD by LHW and professional HCW, through Visitect CD4 LFA, urinary *Mycobacterium tuberculosis* lipoarabinomannan antigen (urine TB LAM) and cryptococcal antigen (CrAg) tests, at different health facility levels.

## Methodology

### Study design

This was a cross-sectional mixed-methods pilot implementation research study within Doctors Without Borders/Médecins Sans Frontières (MSF) supported health facilities in Beira, Mozambique (between 19 April-to-13 July and 14 Oct-to-29 November 2022) and in Kinshasa, Democratic Republic of Congo, DRC (between 14 March-to- 31 August 2022).

### Study background and setting

In Beira, MSF, in collaboration with the ministry of health of Mozambique, has been implementing different catalytic interventions to reduce sexual reproductive health and HIV/AIDS related morbidity, mortality and incidence among key and general population [21]. In Kinshasa, MSF operates a tertiary HIV referral hospital, Centre hopitalier de Kabinda (CHK) with over 50 bed capacity [21].

Frequent break-down of CD4 cell count POC instruments (mainly due to instrument optical challenges and hardware failure), together with low CD4 testing coverage in health facilities, compelled the use of Visitect CD4 LFA. Visitect CD4 LFA was implemented in the activities for scaling up the AHD package of care in Beira at PHCs (Cs de Munhava, Manga Loforte and Inhamuzia) including at a tertiary health facility (PontaGea) and mobile clinic. Similarly, in Kinshasa, the Visitect CD4 LFA was considered for the expansion of the AHD package of care to other public PHCs (St Joseph, St Ambroise, St Clément, CS Mokali) and secondary health facilities (CHK, Luyindu), Table 1.

**Table 1:** Baseline characteristics of study sites.

DRC								
Health facility (and type)	Estimated cohort size (in year 2022)	Patients eligible for CD4 cell count testing in Q1 and Q2 2022^#^				Standard of care (SoC) for CD4 testing at the health facility (2022)	CD4 tests done (SoC), before introduction of Visitect CD4 LFA (Q1 & Q2 of year 2021)	Proposed placement/use of Visitect CD4 LFA including urine TB LAM & CrAg
		High HIV viral load (VL)	New ART initiation	Poor ART adherence	IPD			
CHK (tertiary HIV referral)	2,041	277	232	189	981	3 PIMA instruments (one at IPD and at laboratory) and FACSPresto** (at the laboratory)	1183	IPD and OPD
Luyindu (secondary)	†	†	†	†	274	1 PIMA CD4 instrument	289	IPD and OPD
Biyela (PHC)	495	21	25	†		2 PIMA CD4 instruments (IPD, OPD)	241	OPD
St Clement (PHC)	273	8	32	28		1 PIMA CD4 instrument	119	OPD
Mokali (PHC)	88	4	11	†		1 PIMA CD4 instrument	68	OPD
St Ambroise (PHC)	150	7	12	3		1 PIMA CD4 instrument	74	OPD
**Mozambique**								
**Health facility (and type)**	**Estimated cohort size (in year 2022)**	**Patients eligible for CD4 cell count testing in Q1 and Q2 2022^#^**				**Standard of care (SoC) for CD4 testing at the health facility (2022)**	**CD4 tests done (SoC), before introduction of Visitect CD4 LFA (Q1 & Q2 of year 2021)**	**Proposed placement/use of Visitect CD4 LFA including urine TB LAM & CrAg**
		High HIV VL	New ART initiation	Poor ART adherence				
CS de Munhava health center (PHC)	12,831	262	626	112		2 PIMA CD4 instruments (laboratory)	2560***	OPD
PontaGea (tertiary)*	13,096	1272	761	†		PIMA instruments	1272	OPD and IPD
Manga Loforte (PHC)	3,311	143	361	†		None	Not done (samples referred to testing hubs)^‡^	OPD
Inhamuzua (PHC)	2,223	43	515	†		None	Not done, (samples referred to testing hubs)^‡^	OPD
Mobile clinic	†	†	†	†		None	Not done, (samples referred to testing hubs)^‡^	OPD

Key: ^#^Not all historical data for patients eligible for CD4 testing was available especially for returnees after LTFU and those with no CD4 in the last 12 months.

**FACSPresto is a benchtop laboratory instrument for CD4 cell count testing.

^‡^Hub is a central testing site, and a spoke is a peripheral referral site.

*In this health facility (at the time of this study), there was a limited scope for potential use of Visitect CD4 LFA given the limited support from MSF.

^†^This historical data was not available from facility databases.

***This includes CD4 samples referred from other health facilities to the Munhava health centre laboratory.

IPD In-patient department; OPD Out-patient department.

All the study sites were purposively chosen as they had already ongoing MSF AHD activities including clinical mentorship support.

### Study population

i Patient study population: consenting adult (18-64years) HIV positive patients due for a CD4+ cell count test (for the below mentioned reasons), were offered a CD4 cell count test using the Visitect CD4 LFA.
  - For ART initiation, poor ART adherence, re-engagement after loss-to-follow-up (LTFU), high HIV viral load (VL), no CD4+ cell count in the last 12 months, WHO clinical stage 3 or 4 and providing written informed consent.
ii Frontline HCW were defined as personnel who directly provide medical care and services to patients in the healthcare settings, often serving as the first point of contact within the health system. These include nurses, clinical officers and doctors. Clinical officers are mid-level licenced medical professionals trained to provide a range of healthcare services, often bridging the gaps in areas with shortages of doctors.
iii LHW were defined as personnel who perform functions related to healthcare delivery but do not possess formal professional or paraprofessional education, but received training tailored to their specific role [20]. For this study LHW were counsellors and lay educators involved in patient counselling, tracing and navigation, among other roles.

### Sample size

As the focus of the study was on feasibility, pragmatic sample size estimates were used as opposed to probalistic-statistical sample estimates. Sample size estimation was guided by pragmatic estimates of the expected proportion of patients eligible for CD4 testing in each of the health facilities within 6 months of implementation, Table 1. We expected at least 50% of eligible patients to consent to participation. The reasonable total sample size of 1450 (650 for Mozambique and 800 for DRC) was considered to enable inferential conclusions. Considering this anticipated sample size and other parameters (significance level of 0.05, minimum 10% effect size), the power to test the primary outcome (expected proportion who find it easy to use the 3 tests), detecting a 10% difference from a null proportion of 50%, is 90%. In addition, this sample size estimate was guided by logistical criteria and practical considerations: low shelf-life of Visitect CD4 LFA upon arrival at the study sites (+/-6months), together with the ongoing Covid19 restrictions (during the time of recruitment) and human resource constraints (as most staff were also employed to support other routine program activities). Lastly, our recent published studies in similar settings on the same topic, provide support on the possible adequacy of these pragmatic sample size estimates [21,22].

All patients meeting the criteria were enrolled consecutively to reach the sample size.

### The intervention

In the study sites, existing LHW and frontline HCW who were already involved in HIV related activities, (especially for AHD) at OPD or IPD, were asked to participate in the study. They completed a two-day training that included clarity on why ‘task sharing is being conducted’, performance of POC tests (Visitect CD4 LFA, urine TB LAM and CrAg), and quality assurance to ensure test results are accurate and reliable. Their work roles and responsibilities were broadened to involve task shared AHD POCT for eligible patients.

Venous EDTA blood sample was collected through venipuncture from consenting enrolled patients and tested in Visitect CD4 LFA (AccuBio Limited, Alva, UK), following manufacture instructions. Patients with CD4 cell count less than 200cells per mm^3^ (CD4<200cells/mm^3^) were further tested for cryptococcal antigen, CrAg test (IMMY, Oklahoma, USA), from the remnant EDTA sample, whilst urine was collected for conducting the TB LAM test (Abbott, Illinois, USA). The Visitect CD4 LFA, urine TB LAM and CrAg testing procedures have been described elsewhere [22].

The testers collaboratively determined the distribution of the study workload (phlebotomy, AHD POC testing and quality checks including documentation of activities), based on their immediate work roles and responsibilities. Results from the Visitect CD4 LFA testing were used for patient management. As per national AHD management protocols, patients testing positive for urine TB LAM and or CrAg received appropriate treatment including prophylaxis for those eligible among patients testing negative.

This study’s objective was to assess the feasibility and testing outcomes of LHW and HCW task-shared implementation of AHD POC tests.

### Study hypotheses

We posit that at least 60% of the testers will find it easy to use the AHD POC tests, and this task-shared implementation could potentially increase access to AHD testing among eligible patients, compared to standard-of-care where either a CD4 POC instrument was utilized or where there was no CD4 test conducted at POC.

### Study outcomes

1. Feasibility assessment outcomes:
  1. The primary study outcome was the proportion of testers (LHW and HCW) who found it ‘easy’ to integrate the 3 POC tests within their routine work together with their opinions on the value of task sharing AHD POC tests.
2. Testing outcomes:
  1. Secondary outcomes included measuring the proportion of patients retained at each step of AHD cascade, after an initial CD4<200cells/mm^3^ together with subsequent urine TB LAM and CrAg POC tests. The cascade of testing outcomes was included because it can help to demonstrate the feasibility of sequential AHD POC testing steps that patients undergo, including their retention and this can identify gaps in task shared testing at each stage.
  2. We also examined the average patient waiting time (in minutes) for AHD POC tests and this was defined as the time from sample collection to the communication of the test results to the clinician.

In this study, the diagnostic performance validation of the Visitect CD4 LFA was not conducted as the authors have prior published findings of a multi-country diagnostic performance of Visitect CD4 LFA within the same study settings [22]. Also, Visitect CD4 LFA was registered and approved for routine clinical use in both study countries. There are many other studies that have been published [29,30] however, with varying diagnostic performance of Visitect CD4 LFA.

### Quality assurance in task shared POCT services

All the testers who performed AHD POCT completed a competency training program. All training was performed by qualified individuals (certified laboratory manager) trained by test manufacturers and demonstrated competency. The laboratory manager and senior laboratory technician oversaw the POCT services,

Internal quality control (IQC) testing was regularly done to monitor the accuracy and precision of the POC tests. For CrAg, the manufacturer provides the necessary control materials (positive and negative), whereas for Visitect CD4 LFA and urine TB LAM, there are no manufacture provided controls, and this study utilized remnant patient samples (after testing and clinical care), weekly.

### Qualitative evaluation

At study completion, all the testers (LHW and HCW) were asked to participate in individual self-completed questionnaires (5-point Likert scale and open ended qualitative questions) to explore their opinions on feasibility and integrability of task shared AHD POCT. Only those who gave oral consent were given the questionnaire to complete. Data collection was conducted in Portuguese and French in Beira (Mozambique) and Kinshasa (DRC) respectively. The qualitative data was then translated to English for analysis.

### Data management and ethical approval

Quantitative data was abstracted from study data collection forms into Microsoft Access database. Data analysis was performed using Stata/IC 16.0 (StataCorp, College Station, Texas, USA). Descriptive statistics was used to summarize patient characteristics and testing outcomes. Medians and interquartile ranges (IQR) were used to summarize continuous variables, whereas frequencies and proportions were used for categorical data. Frequencies and proportions were used for the description of the AHD POC testing cascade. Furthermore, a one-sample proportion test was used to assess whether the testers’ ability to integrate AHD POC testing into their routine workflow was significantly different from the 50% expected under the null hypothesis.

Qualitative data from self-completed questionnaires by testers was translated into English. The main discussion points in the transcripts were highlighted and manually grouped into themes, and a coding scheme was created based on the recurrence of the themes.

The study was approved by the MSF ethical review board, ERB (2156), University of Kinshasa, DRC ERB committee (15B/2021), Stellenbosch University health research ethics committee (S23/11/298) and the Mozambique national committee on bioethics for health (85/CNBS/22).

## Results

### Characteristics of patients

Between March to November 2022, a total of 1542 eligible patients were screened for AHD by 35 LHW (34 counsellors, and 1 lay educator) and 45 professional HCW (28 nurses, 9 clinical officers and 8 doctors) in Beira and Kinshasa.

Among the patients tested, majority were female (1005; 65.2%) and overall median age was 36 years [IQR: 26-52], Table 2. The overall prevalence of AHD (CD4<200cells/mm^3^) was 39.2% (604/1542). A total of 960 (62.3%) patients were tested at OPD and the prevalence of AHD was 34% whereas 582 (37.7%) were tested at IPD and prevalence of AHD was 47%.

**Table 2:** Characteristics of patient study patients.

Variable	Proportion n (%)
**Total**	1542
**Country**	
Mozambique	699 (45.3)
DRC	843 (54.7)
**Sex**	
Male	530 (34.4)
Female	1005 (65.2)
Unknown	7 (0.5)
**Median age (years) [IQR]**	36 [26 - 52]
**Reason for CD4 testing^#^**	
ART initiation	776 (50.3)
High HIV VL	191 (12.4)
Re-LTFU	131 (8.5)
Hospital admission*	352 (22.8)
Missing CD4^†^	89 (5.8)
Other	3 (0.2)
**Visitect LFA CD4 ≤ 200cells/mm^3^**	604 (39.2)
**Urine TB LAM**	
Positive	146 (24.2)
Negative	341 (56.5)
Missing^‡^	117 (19.4)
**Plasma CrAg**	
Positive	22 (3.6)
Negative	415 (68.7)
Missing**	167 (27.6)

Key:

^#^These are not mutually exclusive (*for example, some patients who were ‘admitted at IPD’ had high HIV VL and/or were initiating ART*)

*Admitted HIV positive patients are systematically tested for CD4 cell count

^†^No CD4 test ever done in the last 12months

^‡^Urine TB LAM tests not done (or not documented if they were done) among patients with CD4 < 200cells/mm^3^

**CrAg tests not done (or not documented if they were done) among patients with CD4 < 200cells/mm^3^

### Primary outcomes

#### Characteristics of testers and feasibility of task shared POC testing.

Of the 80 testers (35 LHW and 45 professional HCW), majority (49/80; 61.3%) were from Beira, Table 3. Professional HCW had diploma or degree qualification, whereas LHW (counsellors and lay educator) had a one-to-two week/s on-job training for their primary roles (Table 3). The primary roles of LHW involved HIV testing and counselling (for HIV, ART, pre-and post-exposure prophylaxis), but all had experience in conducting rapid POC testing (beyond HIV).

**Table 3:** Characteristics of AHD POC testers.

Country	Primary work role (total)	Education/ qualification	Classification	Median years in service provision	Experience in conducting other POC tests (aggregated)	General duties (aggregated)
Mozambique	Counsellors (23)	2 week on-job training	LHW	3	Rapid tests (HIV, pregnancy, malaria, pregnancy)	HIV counselling, testing, prevention, patient registration, ART reception and refill, ART enhanced adherence counselling, AHD counselling, patient follow-up, PrEP counselling.
	Lay educator (1)	1-2 weeks on-job training	LHW	2	Rapid test (HIV, malaria)	Treatment literacy activities, HIV counselling and testing, index testing case finding and PrEP counselling. Organizing of patient files, patient follow-up.
	Nurse (17)	Diploma	Professional HCW	10	Rapid tests (HIV, syphilis, Hepatitis, malaria) glucometers	HIV counselling and testing, patient registration, ART initiation and refills, prenatal care, HIV and syphilis testing, patient follow-up.
	Clinical officers (6)	Diploma	Professional HCW	2	Rapid tests (HIV, syphilis, Hepatitis), PIMA CD4	Clinical care, ART initiation, screening and management (national TB program), ART follow-up, HIV testing and counselling. ART and TB consultation.
	Doctors (2)	Degree	Professional HCW	2	Rapid tests (HIV, Syphilis, malaria, hepatitis)	Clinical care, ART initiation, screening and management (national TB program), ART follow-up, HIV testing and counselling. ART and TB consultation. Team coordination.
DRC	Counsellors (11)	On job training	LHW	2	Rapid tests (HIV, pregnancy), glucometer	HIV counselling, testing, prevention, patient registration and ART reception, ART adherence counselling, AHD counselling, patient follow-up, PrEP counselling.
	Nurses (11)	Diploma	Professional HCW	7	Rapid tests (HIV, Syphilis, hepatitis), glucometers, PIMA CD4	HIV testing, counselling, patient registration, ART initiation and refills, prenatal care, HIV and syphilis testing, patient follow-up.
	Clinical officers (2)	Diploma	Professional HCW	4	Rapid tests (HIV, Syphilis, SARSCov2), PIMA CD4	Clinical care including patient consultation for HIV, TB and other opportunistic infections including follow-ups. Team coordination and supervision.
	Doctors (7)	Degree	Professional HCW	4	Rapid tests (HIV, syphilis, SARSCov2), PIMA CD4	Clinical care including patient consultation for HIV, TB and other opportunistic infections including follow-ups. Team coordination and supervision.

Majority (66/80; 82.5%) of the testers conducted AHD POCT from OPD within PHC, secondary as well as tertiary health facilities; however, only few conducted testing at IPD (14/80; 17.5%) and these were mostly in CHK tertiary hospital (Table 4).

**Table 4:** Patients screened for AHD per health facility tier level in all study sites.

Study site tier levels (number of health facilities)	Number of patients tested for CD4 cell count (%)
PHC (8)	704 (45.7)
Secondary (1)	90 (5.8)
Tertiary (2)	748 (48.5)

During the study period (5–6 months), the median number of tests conducted by counsellors within OPD settings was 27 [IQR: 16–34], whereas nurses, clinical officers together with doctors conducted a median of 19 and 11 tests respectively. There were no notable differences in AHD POC test procedural performances between LHW and HCW at all the study health facilities.

#### Perceived value of task sharing and integrability of the AHD POC tests.

From the 80 testers, only 42 (52.5%) consented and attempted the self-completed questionnaire. The qualitative study findings were synthesized into two primary themes which include: (1) perception of task shared POC testing for AHD and (2) impact on service delivery.

#### Theme 1: Perception of task shared POC testing for AHD.

Most of the 42 testers perceived value in task-shared AHD POCT, describing it as an approach that expands testing access and enables more patients to be tested quickly and efficiently at the POC. Respondents emphasized that task-shared POC testing for AHD can complement instrument and laboratory-based testing while fostering teamwork and shared responsibility within healthcare settings. Quotes below, describe some of these tester opinions:

HCW in a tertiary health facility in DRC “*this (AHD POC testing) improves our capacity to assess and manage patients with AHD appropriately at POC*”. Male, 38 years.

LHW in a secondary health facility in DRC “*on days when I am overwhelmed with other duties and not able to conduct these tests (AHD POC), I can always ask the patient to go get tested at the laboratory*”. Female, 32 years.

LHW in a PHC facility in Beira “*patient counselling is my activity, but I enjoy having to also conduct testing even though it is not supposed to be my work*, *I am just happy to learn new skills*”. Female, 29 years.

A doctor in Mozambique highlighted that with their high workload, performing POC testing will better be delegated to LHW.

“*Whilst performing these POC tests can help with quick results, sometimes it can take time away from other responsibilities needing my expertise especially managing complicated patients*”. Male, 44 years.

One LHW participant in a PHC in Beira highlighted the need for better remuneration given the extra workload.

“*sometimes I feel privileged handling new tasks and sometimes too I feel stressed because the workload is too much, yet my salary is the same. This is too much work, and I get very tired sometimes”.* Female, 34 years.

#### Theme 2: Impact on service delivery.

In the opinion of HCW, use of POC tests with immediate issuance of results could improve patient satisfaction and convenience for AHD diagnostics and services as illustrated by the below quotes.

One respondent from a PHC facility that usually referred patients for CD4 testing to a hub facility noted that “*POC testing has the potential to ensure that only patients who have severe conditions are sent to higher-level care”.* Female, 39 years.

A HCW recounted one patient explaining how POCT makes his life less difficult as he does not have to go to the laboratory for testing where he may join a long queue and must come back for further tests.

A LHW in Mozambique described that patients were willing to wait for their POC AHD results as well as the perceived impact of POC testing services:

*“true POC testing for CD4 can help improve satisfaction of patients for AHD services as it allows decentralization of diagnostics. This reduces patient travel and wait times, improves timely diagnosis and treatment initiation”.* Male, 35 years.

Of the 42/80 (52.5%) testers who successfully managed to complete the self-administered questionnaire, 64% (27/42) reported it was easy/very-easy to conduct the AHD POC tests, and 59% (25/42)—mostly LHW (67%)—found integrating these tests into their routine workflow easy. In contrast, nearly all clinical officers and doctors who completed the self-administered questionnaire (8/12; 66.7%) indicated that integrating POC testing into their daily work was difficult. The proportion of testers who reported managing to integrate AHD POC testing into their routine workflow was significantly higher than 50% expected under the null hypothesis (p = 0.032, one-sample proportion test) (Table 5).

**Table 5:** Self-completed questions on tester perceptions on use of the AHD POC tests.

	Very difficult	Difficult	Neutral	Easy	Very easy	Total
Rate your ability to conduct the Visitect CD4 test together with urine TB LAM and plasma CrAg	5 (11.9%)	7 (16.7%)	3 (7.1%)	20 (47.6%)	7 (16.7%)	42
Rate your ability to integrate Visitect CD4 LFA, TB-LAM & CrAg into your daily work	6 (14.3%)	8 (19.0%)	3 (7.1%)	19 (45.2%)	6 (14.3%)	42

### Secondary outcomes

The use of Visitect CD4 LFA at CHK (DRC) and Munhava (Mozambique) health facilities, resulted in 608 and 342 patients tested respectively, Table 6. Alongside the existing standard of care for CD4 testing instruments, the use of Visitect CD4 LFA contributed to increasing the proportion of patients receiving CD4 testing by 10.7% in Munhava (Beira) and 22.9% in CHK (DRC).

**Table 6:** Visitect CD4 LFA tests conducted in 2 health facilities compared to standard of care CD4 testing.

Country	Health facility	CD4 tests done in SoC 2021 (Q1 & Q2 2021)	CD4 tests done in SoC 2022 (Q1 & Q2 2022)	CD4 tests done in Visitect CD4 LFA (March-Nov 2022)
DRC	CHK	1183	1370	608 (22.9%)*
Mozambique	Munhava	2560	1905	342 (10.7%)*

Key: *Visitect CD4 tests done, as a percentage of all CD4 tests done (including SoC) in the time-period (March – November 2022)

#### Cascade of AHD testing.

**Fig 1:**
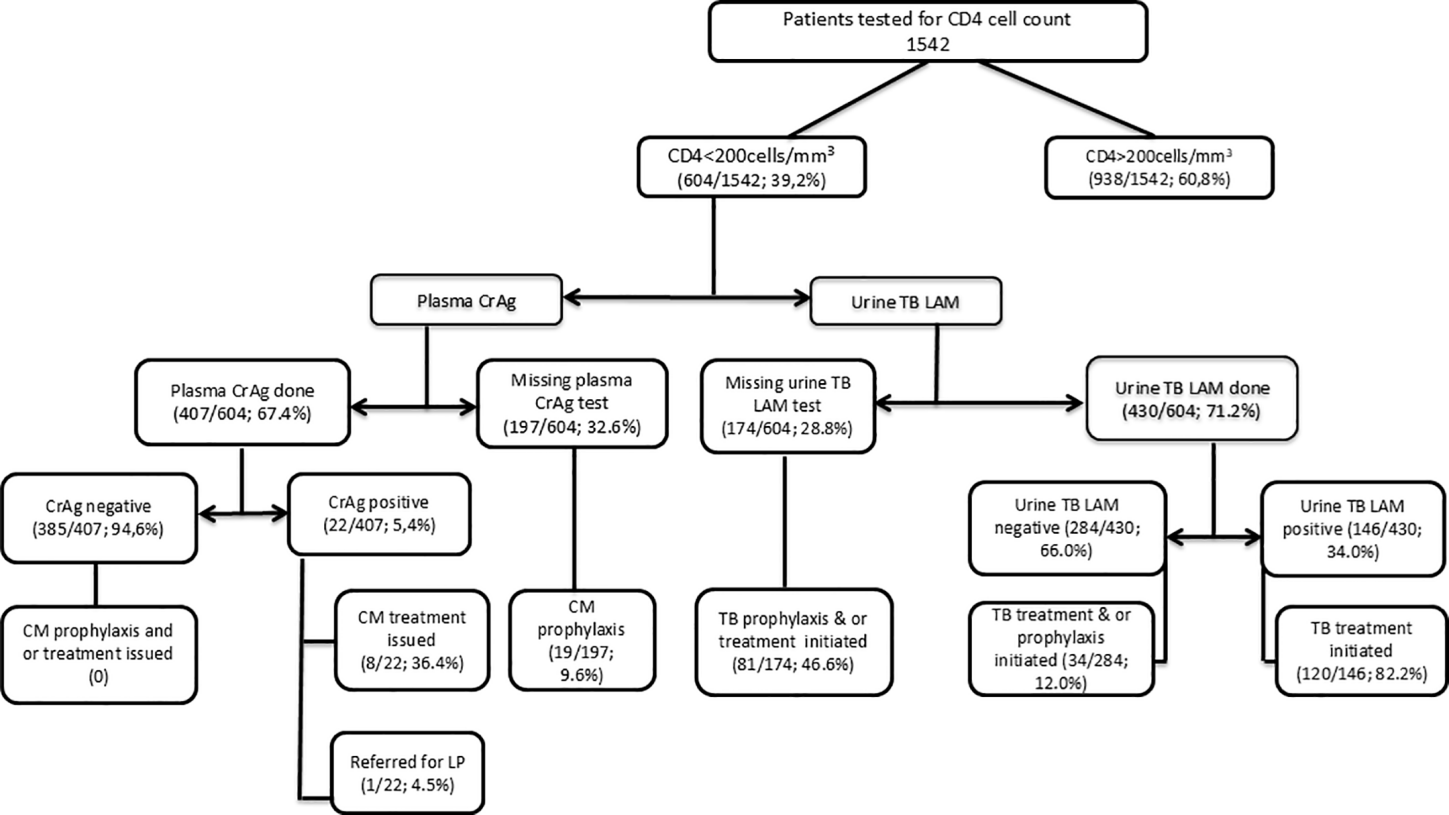
Overall cascade of AHD POC testing in Mozambique and DRC for patients with CD4 less than 200cells/mm^3^. Key: LP, Lumbar puncture; CM, Cryptococcal meningitis.

Among the 604 participants with CD4 < 200cells/mm^3^ (in all study sites), 71.2% (430) and 67.4% (407) had a documented urine TB LAM and plasma CrAg test results, respectively (Fig 1). Among these, a total of 146/430 (34.0%) tested positive for urine TB LAM and 82.2% (120/146) had a documented TB therapeutic intervention whereas 22/407 (5.4%) tested positive for plasma CrAg and 36.4% (8/22) had documented therapeutic intervention.

Majority of study participants with un-documented urine TB LAM results were from Munhava health facility (60/107; 56.1%) followed by CHK (58/67; 86.6%) and Ponta-Gea hospital (24/107; 22.4%).

#### Turn-around time for AHD POC testing results.

A total of 801/1542 (51.9%) patients had a recorded time for sample collection to completion of testing and communication of results to clinician. Among these, 76.3% (611/801) patients with CD4 cell counts greater than 200cells/mm^3^, had a median time for completing the Visitect CD4 test and conveying the results to the clinician of 59 minutes [IQR: 48–71]. However, among patients with a CD4 < 200cells/mm^3^, only 23.7% (190/801) had a recorded time flow for urine TB LAM and CrAg results and the median time for the total flow was 79 minutes [IQR: 66–102]. Median turn-around times were similar between LHW and HCW testing.

## Discussion

This implementation study demonstrates that task-sharing the implementation of AHD POC tests between LHW and frontline professional HCW is feasible at primary and secondary healthcare levels. However, this study also highlights that incorporating task-shared testing into the regular clinical practices of these cadres can be a challenging process that requires careful planning, especially regarding data documentation and quality assurance.

For this study, rather than recruiting new staff, work-roles and responsibilities for existing LHW and frontline HCW already involved in AHD activities, were reviewed and broadened to include task shared AHD POCT. Nearly fifty percent of the testers were LHW whose primary roles mainly included counselling, testing and follow-up of PWH. Study findings from self-completed questionnaires point towards LHW having the better mix of tasks with AHD POC tests compared to professional HCW. Many LHW (66.7%) expressed their ability to integrate AHD POC testing within their routine workflow whilst most clinical officers expressed difficulty to integrate POCT. Similar findings have been reported in other studies and WHO recommendations that have also highlighted that LHW are well placed to perform POC testing [4,31] as compared to clinical officers who have less availability for additional tasks relative to other cadres. As other HCWs are equally capable of providing HIV testing and counselling information (for HIV, ART, pre-and post-exposure prophylaxis) as shown in other studies [32,33], LHW primarily specific for HIV testing services could possibly have their scope broadened to include AHD POC testing. However, roles for LHW require on-going evaluation especially as patient needs are evolving and generally, POC testing should not be merely shifted away to LHW, rather, it must be implemented with clarity on their other work roles along with POCT training and ongoing supervision.

Along-side instrument based CD4 testing standard-of-care, task-shared POC testing contributed to a higher overall number of patients tested (10.7% in Beira and 22.9% in Kinshasa) with immediate availability of results. The median time for completing the Visitect CD4 LFA test and conveying the results to the clinician was 59 minutes. Within the complexities of real-world engagement in HIV services, especially as the standard-of-care for CD4 testing for many PHCs involves referral of samples or patients to testing hubs, task shared AHD POCT could save the patient time and cost for repeated travel to the clinic for AHD screening results and/or therapeutic as well as psychosocial interventions. This could minimize attrition, morbidity and or mortality as echoed by other studies [12,34]. It is not surprising that majority of testers who attempted the self-completed questionnaire perceived the value of this complementary approach of task-shared AHD POC testing in bridging existing barriers for immediate access to AHD POC testing, especially in PHCs. Prior research highlights that the greatest benefits from task sharing could be more pronounced in primary and secondary care settings [4,35]. At PHC, task shared/shifted POCT with LHWs can significantly expand access to timely diagnostics and reduce unnecessary referrals. Secondary clinics also benefit as task sharing alleviates workload on scarce HCW, enabling quicker patient throughput and improved linkage to care. In contrast, tertiary facilities, which have more specialized staff and laboratory capacity, may see relatively less efficiency gains from task sharing, though some decongestion effects remain valuable.

Critical to the success of task shared AHD POCT is effective user training and ongoing monitoring support to ensure test results are accurate, particularly as studies are demonstrating varying reliability of Visitect CD4 LFA [29,30]. It is essential for the manufacturer to optimize the accuracy of the Visitect CD4 LFA and perhaps elongate the shelf-life to at least 18 months. Nonetheless, the two-day user-training in this study for the three AHD POC tests, significantly enhanced mastery of testing skills, particularly to ensure that the precise multi-stage manipulation of Visitect CD4 LFA is accurately adhered to. Generally, decentralized health facilities with no laboratories could elect a POCT coordinator who should be responsible for all aspects of POCT performed, in line with other research [36]. To help maintain the trueness of POCT results, hub laboratories could regularly send ‘blinded’ testing samples (external quality assessment; EQA) to decentralized health facilities for testing.

Among patients with AHD, 28.8% and 32.6% were not recorded in the study register as having undergone urine TB LAM and CrAg POC tests, respectively, and it is unknown whether these patients were already on therapeutic intervention for TB and or cryptococcal meningitis, before the study. Most of these patients were from facilities with high patient volumes (CHK and Munhava). In line with other research [37], our study indicates that data registration proved difficult due to complex patient and register flows in health facilities, fragmented responsibility and limited supervision. This underscores possible implementation challenges in real-world scenarios with near-similar or more imperfect set-up. Some studies highlight the importance of resilient teamwork in task sharing to minimize fragmented tasks and poor responsibility [38,39] and other studies emphasise that complete task shifting can instil a sense of more responsibility than task sharing [40]. Nevertheless, prior to the adoption of task shared POCT, it is recommended that a needs analysis and critical evaluation of patient flows, mix of HCW tasks and data documentation plans, be performed [41].

In health facilities without laboratories (especially PHC), POCT users are often nurses and other clinical staff members with existing patient care responsibilities. Generally, the battery of POC tests to be conducted for a newly diagnosed HIV patient due for AHD screening, usually include three confirmatory HIV rapid tests, CD4 cell count, urine TB LAM and CrAg, and all these could take 2 hours to complete, especially for a patient with CD4 < 200cells/mm^3^. To minimize under-utilization of such innovative POC tests especially in decentralized health facilities, national health policy and strategy reforms are needed to support formalization of task shared/shifted integrated POCT [28]. National health strategic plans and policies for human resources in health, including vertical disease implementation manuals, should emphasize the transversal role that LHW could play in integrated POC disease testing (even beyond AHD) [28]. Clarity on the range of POC tests that can be task shifted to LHW, together with establishment of in-country formal training services for POCT including framework for ongoing support to task-shifted testing services, are needed. Leveraging practices with evidence of effectiveness is increasingly important, as countries and partners work together within a shrinking fiscal landscape.

Task shifting has the potential to also advance global health priorities like universal health coverage and health security, as emphasized by the 2023 World Health Assembly resolution on strengthening access to quality diagnostics [42].

Strengths of this study include multi-country implementation and a relatively large sample size. Limitations of the study include the stock-outs of Visitect CD4 LFA tests (for almost 3 months) due to importation delays. Due to staff turnover and frequent department rotations in the health facilities, nearly half of the testers did not manage to attempt the self-administered questionnaire, especially at tertiary health facilities. Many facilities had missing historical data and this limited study data analysis and generalizability of findings. Also, the study took place in MSF supported health facilities, which are usually better resourced, which could be a challenge for some national programs. Nonetheless, studies are required to explore the experiences of patients along task shared AHD diagnostic pathway.

## Conclusion

Task shared AHD POC testing is feasible and integrable within the routine work of LHW and frontline HCW and it can strengthen earlier identification patients with AHD and potentially reduce morbidity and mortality. However, task sharing requires teamwork as documentation of testing activities can increase in complexity as testing responsibilities become shared among multiple health care professionals. Nevertheless, LHW are well suited for POC testing due to limited availability and higher clinical workload of other HCW. These findings can inform country-level considerations about the prospects of task shared POCT for AHD and other priority diseases.

## Supporting information

S1 File Inclusivity-in-global-research-questionnaire. (DOCX)

## Abbreviations

AHD: Advanced HIV disease
ART: Antiretroviral Therapy
DRC: Democratic Republic of Congo
CrAg: Cryptococcal Antigen
HCW: Health Care Workers
IPD: In-patient department
LFA: Lateral Flow Assay
LHW: Lay Health Workers
LTFU: Loss-to-follow-up
MSF: Médecins Sans Frontières
OPD: Out-patient department
PHC: Primary Health Care
POC: Point of Care
POCT: Point-of-care testing
TB-LAM: Urinary Mycobacterium Tuberculosis Lipoarabinomannan antigen.
