# Supplementary material for: Feasibility and testing outcomes of task-shared implementation of advanced HIV disease point of care tests in Beira (Mozambique) and Kinshasa (DRC)
Source: PLoS One. 2026 Feb 9;21(2):e0339469. doi: 10.1371/journal.pone.0339469 (PMC12885292; doi:10.1371/journal.pone.0339469)
Supplement: S1 File — (DOCX) [file pone.0339469.s001.docx]

Inclusivity in global research

PLOS’ policy on inclusivity in global research aims to improve transparency in the reporting of research performed outside of researchers’ own country or community and ensures that PLOS publications reporting global research adhere to high standards for research ethics and authorship. Authors of relevant research articles may be asked to complete the questionnaire below, which outlines ethical, cultural, and scientific considerations specific to inclusivity in global research. This questionnaire may be requested when researchers have travelled to a different country to conduct research, if research uses samples collected in another country, research with Indigenous populations or their lands, or if research is on cultural artefacts. Researchers travelling to another country solely to use laboratory equipment will not normally be required to complete the questionnaire. However, the questionnaire can be requested at the journal’s discretion for any submission – if you have been requested to complete this questionnaire by the PLOS journal you submitted to, please do so.

Please complete the questionnaire below and include this as a Supporting Information file with your manuscript. Note that if your paper is accepted for publication, this checklist will be published with your article in the supporting information files. Please ensure that you reference the checklist in the main body of your manuscript. We suggest adding a subsection ‘Inclusivity in global research’ to your Methods section and adding the following sentence: “Additional information regarding the ethical, cultural, and scientific considerations specific to inclusivity in global research is included in the Supporting Information (SX Checklist)”

The questions have been designed to be applicable to a wide range of study types, and there are subsections for both human subjects research and non-human subjects research. If any of the questions are not relevant to your research please mark them as “N/A” as appropriate.

**Ethical considerations, permits and authorship**

*This section is applicable to all research types.*

Provide details as to who granted permissions and/or consent for the study to take place in the Methods section of your manuscript. This should include the names of **all** ethics boards, governmental organizations, community leaders or other bodies that provided approval for the study. If individuals provided approval refer to these people by their role or title but do not list their name(s).

Reported on page number: page 11, line 293-to-295. The study was approved by the MSF ethical review board, ERB (2156), University of Kinshasa, DRC ERB committee (15B/2021), Stellenbosch University health research ethics committee (S23/11/298) and the Mozambique national committee on bioethics for health (85/CNBS/22).

If there were any deviations from the study protocol after approval was obtained please provide details of these changes in the Methods section of your manuscript.

Reported on page number: There were no deviations from the study protocol after approvals.

Did this study involve local collaborators that are residents of the country where the research was conducted or members of the community studied? If you do not have any authors from said communities, please provide an explanation for this below.

Yes. For Democratic Republic of Congo, Richard Ingwe Chuy, Aimé Mboyo are local collaborators from the national HIV/AIDS and STI Control program of the Democratic Republic of Congo. In Mozambique, Pedro David Manusso is a local collaborator from the national ministry of health in Mozambique.

Everyone listed as an author should meet PLOS’ criteria for authorship and all individuals who meet these criteria should be included in the author byline, rather than the acknowledgements. For further information please see the journal’s Authorship Policy.

**Human subjects research (e.g. health research, medical research, cross-cultural psychology)**

Did you obtain written informed consent from a representative of the local community or region before the research took place? How did you establish who speaks for the community? Details of written informed consent obtained from study participants should be reported separately in the Methods section of your manuscript.

Our research involved obtaining written informed consent directly from individual HIV positive patients for sample collection, and oral consent from healthcare workers implementing the testing. These procedures were approved by the relevant institutional and national ethical review boards prior to study initiation. The study context, setting, and design did not require written consent from a community or regional representative as a formal prerequisite. Instead, the “community” is understood as the patients and healthcare workers directly participating, with oversight by institutional review boards ensuring community protection in line with local norms and regulation.

How did members of the local community provide input on the aims of the research investigation, its methodology, and its anticipated outcome(s)?

In preparing the informed consent documents and other study materials, we prioritized clarity and accessibility to ensure understanding by the stakeholders directly involved in the study. Although our study did not specifically engage the broader local community, we conducted pilot testing of the consent forms and procedures with the intended health care workers and patients. Feedback from this pilot phase helped us refine the language and presentation of the consent documents to be clear, culturally appropriate, and suitable for the literacy levels of these participants. Written consent forms were designed in the local language, and study staff were trained to provide thorough verbal explanations and answer questions to ensure study participant understanding.

When engaging with the local community, how did you ensure that the informed consent documents and other materials could be understood by local stakeholders?

During the design and planning of the study, formal input from members of the local community was not sought. However, the research team engaged directly with healthcare workers and lay health cadres who serve the community, obtaining their oral consent and feedback regarding the feasibility and acceptability of POC testing as part of task sharing. The study protocol, including the approach to patient consent for testing, was developed in alignment with standard ethical guidelines as well as had local and international institutional review board approvals. The study outcomes of the research will improve access to advanced HIV disease care in the community and these finding are planned to be shared with local stakeholders, fostering future community engagement.

Will the findings of the research be made available in an understandable format to stakeholders in the community where the study was conducted (e.g. via a presentation, summary report, copies of publications, etc.)? Please provide details of how this will be achieved.

Yes, the findings of the study will be disseminated in accessible and appropriate formats to the community stakeholders involved in the research. Specifically, we plan to:

1. Organize feedback sessions at the health facilities where the study was conducted, targeting healthcare workers, lay health cadres, and community representatives to share key results and discuss implications in an understandable way.
2. Prepare a plain-language summary report highlighting the main findings, recommendations, and next steps, which will be distributed to participating clinics and local health authorities.
3. Provide copies of the published manuscript and any related policy briefs or guidelines developed as part of the study to relevant stakeholders upon request.
4. Engage with local health leadership and community advisory boards to ensure ongoing dialogue and incorporate their perspectives in future research or implementation efforts.

**Non-human subjects research using specimens/ animals collected as part of the study, or those housed in archival collections. Examples include archaeology, paleontology, botany and zoology.**

Did the permission you obtained from a local authority to perform the study include an agreement on access to outputs and benefit sharing? This may include procedures to enable fair distribution of the benefits and resources arising from the research performed. Please include any details of Prior Informed Consent and Benefit Sharing Agreements obtained. These may be required by field-specific regulations, for example the Convention on Biological Diversity (CBD) and the associated Nagoya Protocol.

The local authority’s approval included oversight of ethical conduct, patient confidentiality, and community welfare, but did not explicitly detail formal benefit-sharing agreements under frameworks such as the Convention on Biological Diversity (CBD) or the Nagoya Protocol, as the study did not involve genetic resources or bioprospecting.

Nonetheless, we are committed to ethical research practices including: ensuring equitable access to study findings by sharing results with participating health facilities and stakeholders through reports, presentations, and publications. If relevant, any future utilization of biological samples or data for secondary research would be governed by separate agreements compliant with local and international regulations

If the material used in your study was imported, please A) provide the year it was imported and B) indicate whether permits were obtained to import/export the materials used, C) provide details of any permits obtained. If this information is not available, please indicate this.

No study marterial was imported or exported.

If you used archival specimens, please state how the material used in your study was acquired by the institute it is held in and provide details of any permits obtained for the original excavations/ sample collection. If this information is not available, please indicate this.

No archived study marterial was used.

How was the potential cultural significance of the materials collected in your study to local communities considered in your research design? Were Indigenous peoples and/or local researchers and institutions involved with archaeological excavations / collection of specimens? If so, please provide a description of their involvement.

Our research focused on biological sampling for clinical diagnostic purposes and did not involve Indigenous peoples specifically or any archaeological excavations or culturally sensitive specimens. Accordingly, Indigenous peoples or local institutions related to cultural heritage or archaeology were not involved in the collection of materials.

If your manuscript includes photographs of human remains please indicate whether authors obtained permission from descendants or affiliated cultural communities to do so.

Our research does not include any photographs of human remains.
